# Supplementary material for: Mendel, a foundation model of human genetic variation, prioritizes regulatory variants and improves gene expression prediction
Source: bioRxiv. 2026 Sep 30:2026.09.28.755183. Preprint. [Version 1] doi: 10.64898/2026.09.28.755183 (PMC13648636; doi:10.64898/2026.09.28.755183)
Supplement: Supplementary file 1 [file media-1.pdf]

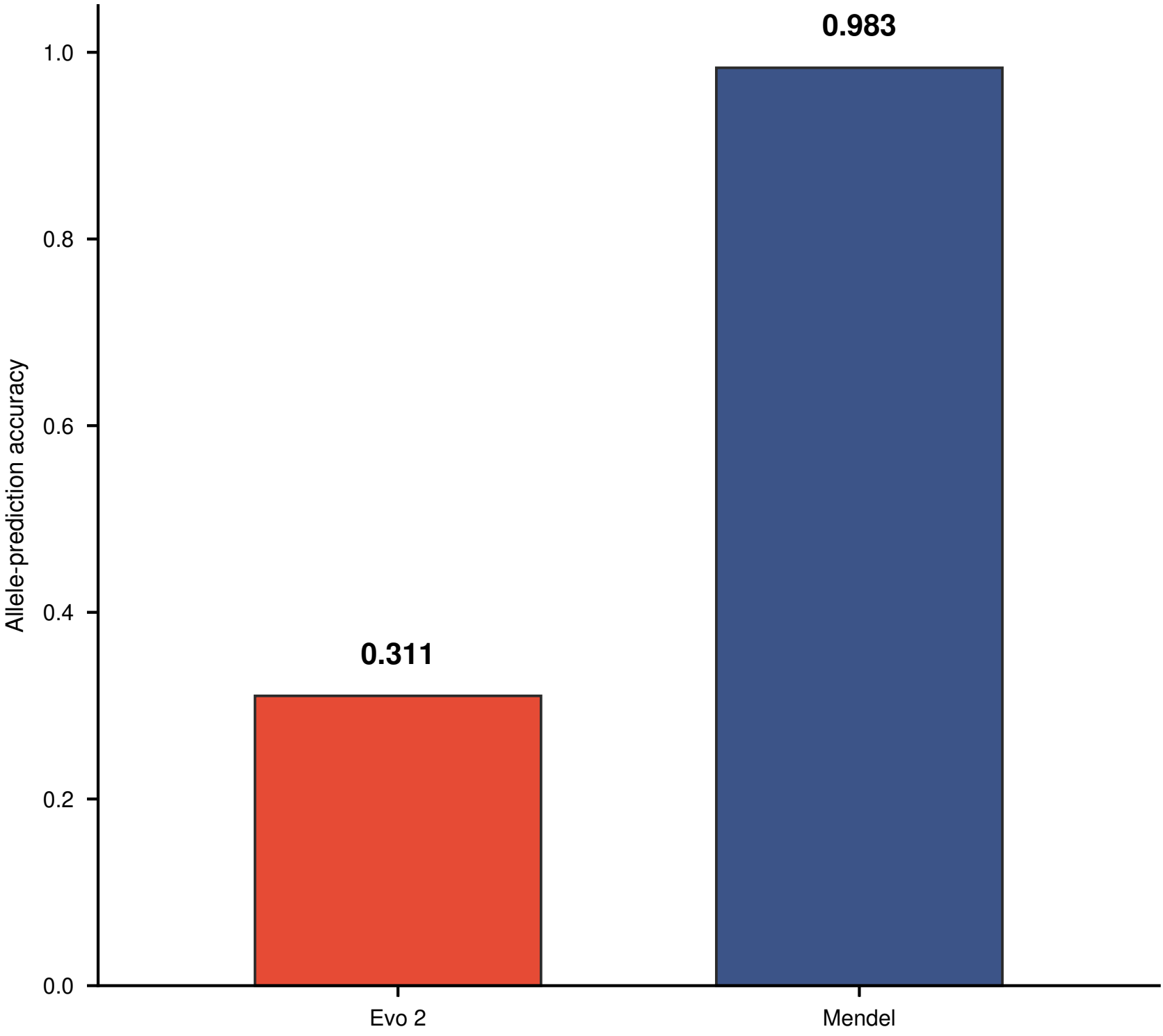
0.983
0.311
1.0
0.8
0.6
0.4
0.2
0.0
Allele-prediction accuracy
Evo 2
Mendel
