# Supplementary material for: Mendel, a foundation model of human genetic variation, prioritizes regulatory variants and improves gene expression prediction
Source: bioRxiv. 2026 Sep 30:2026.09.28.755183. Preprint. [Version 1] doi: 10.64898/2026.09.28.755183 (PMC13648636; doi:10.64898/2026.09.28.755183)
Supplement: Supplementary file 2 [file media-2.pdf]

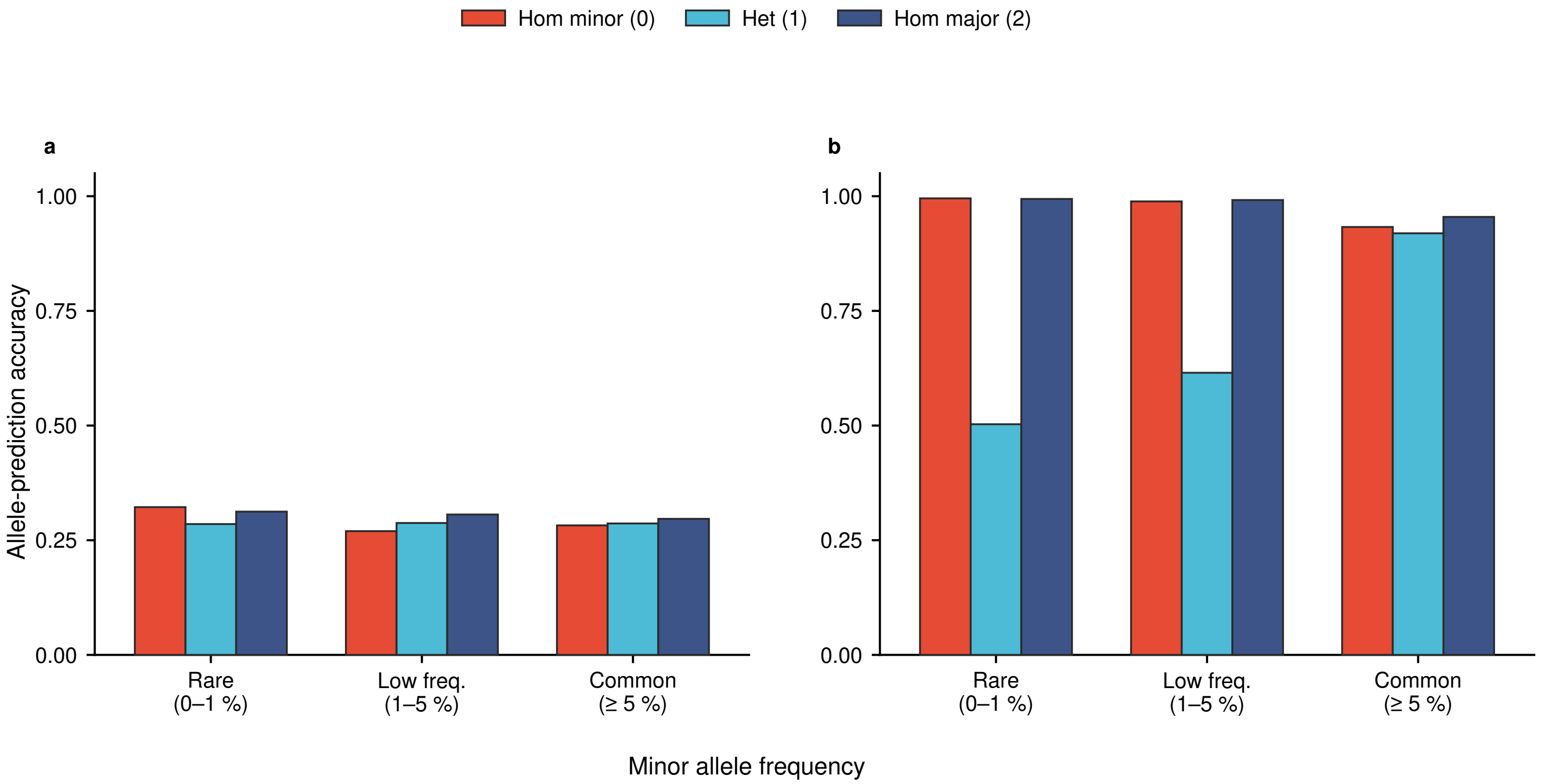
Hom minor (0)
Het (1)
Hom major (2)
a
b
Allele-prediction accuracy
1.00
0.75
0.50
0.25
0.00
Rare
(0–1 %)
Low freq.
(1–5 %)
Common
(≥ 5 %)
Minor allele frequency
