# Supplementary material for: Mendel, a foundation model of human genetic variation, prioritizes regulatory variants and improves gene expression prediction
Source: bioRxiv. 2026 Sep 30:2026.09.28.755183. Preprint. [Version 1] doi: 10.64898/2026.09.28.755183 (PMC13648636; doi:10.64898/2026.09.28.755183)
Supplement: Supplementary file 3 [file media-3.pdf]

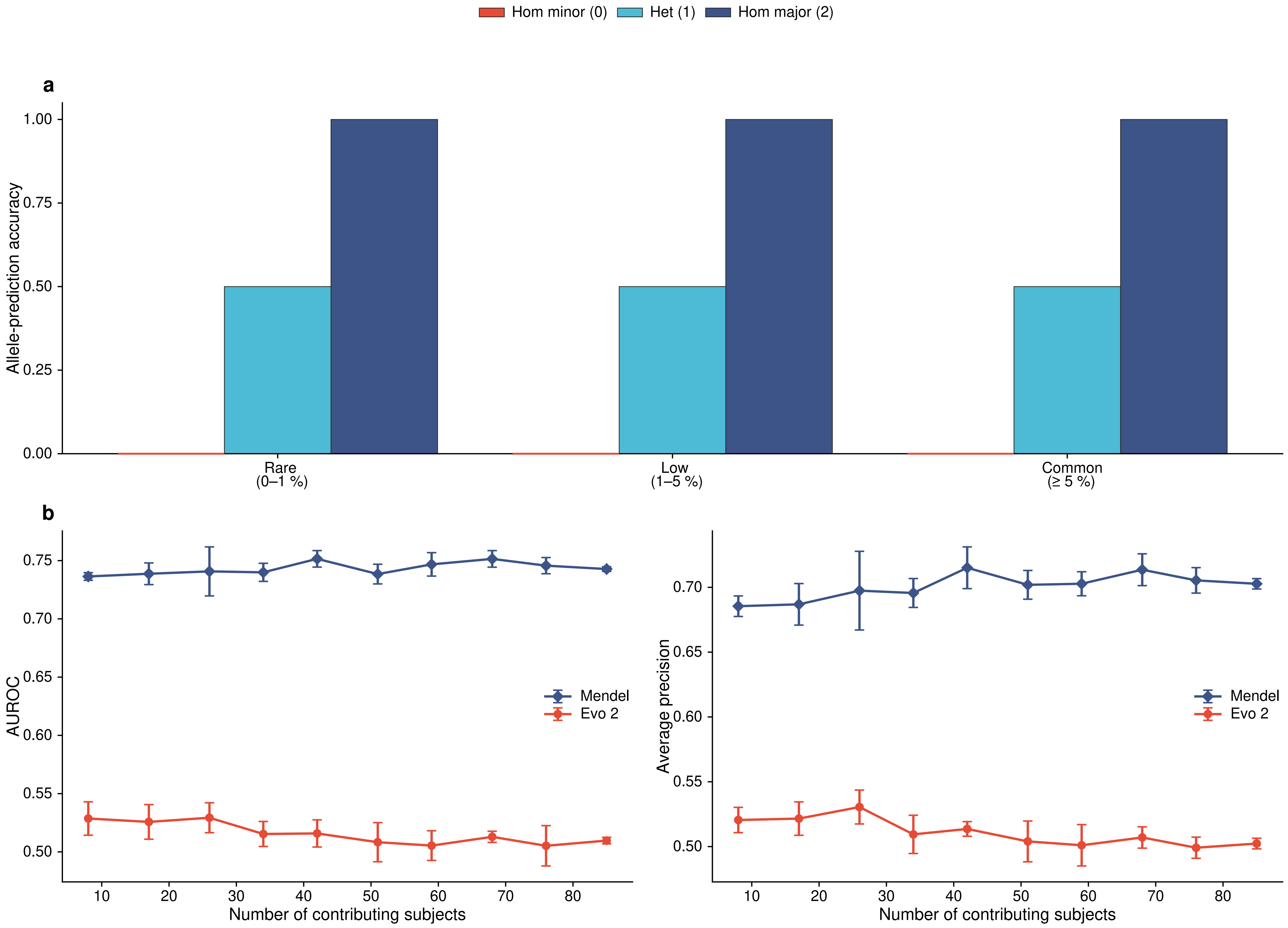

Hom minor (0)
Het (1)
Hom major (2)
a
Allele-prediction accuracy
1.00
0.75
0.50
0.25
0.00
Rare
(0–1 %)
Low
(1–5 %)
Common
(≥ 5 %)
b
AUROC
0.75
0.70
0.65
0.60
0.55
0.50
Mendel
Evo 2
10
20
30
40
50
60
70
80
Number of contributing subjects
Average precision
0.70
0.65
0.60
0.55
0.50
Mendel
Evo 2
10
20
30
40
50
60
70
80
Number of contributing subjects
