# Supplementary material for: Mendel, a foundation model of human genetic variation, prioritizes regulatory variants and improves gene expression prediction
Source: bioRxiv. 2026 Sep 30:2026.09.28.755183. Preprint. [Version 1] doi: 10.64898/2026.09.28.755183 (PMC13648636; doi:10.64898/2026.09.28.755183)
Supplement: Supplementary file 4 [file media-4.pdf]

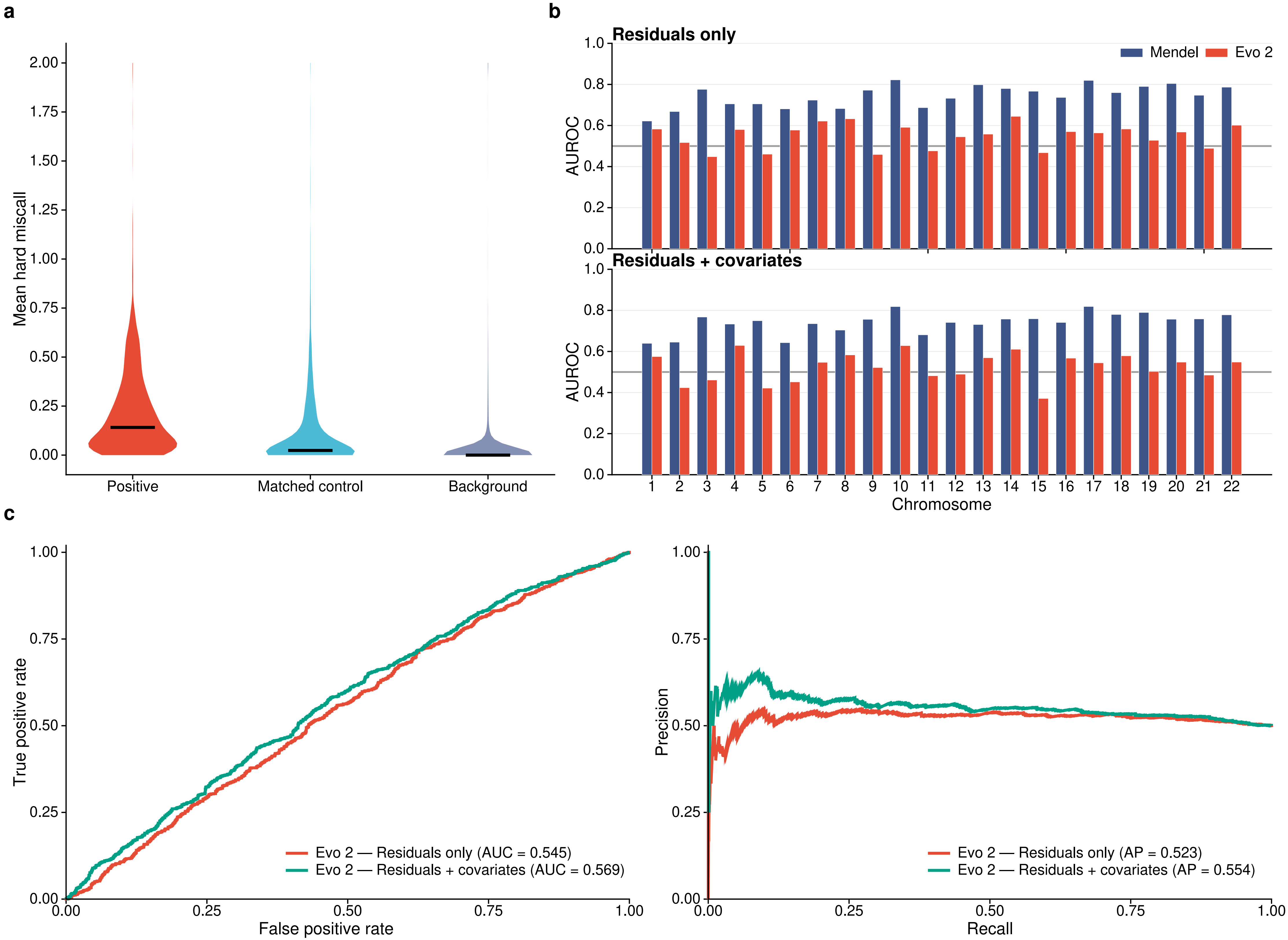

a
Mean hard miscall
2.00
1.75
1.50
1.25
1.00
0.75
0.50
0.25
0.00
Positive
Matched control
Background
b
Residuals only
Mendel
Evo 2
AUROC
1.0
0.8
0.6
0.4
0.2
0.0
Residuals + covariates
AUROC
1 2 3 4 5 6 7 8 9 10 11 12 13 14 15 16 17 18 19 20 21 22
Chromosome
c
True positive rate
False positive rate
0.00
0.25
0.50
0.75
1.00
Evo 2 — Residuals only (AUC = 0.545)
Evo 2 — Residuals + covariates (AUC = 0.569)
Precision
Recall
Evo 2 — Residuals only (AP = 0.523)
Evo 2 — Residuals + covariates (AP = 0.554)
