# Supplementary material for: Mendel, a foundation model of human genetic variation, prioritizes regulatory variants and improves gene expression prediction
Source: bioRxiv. 2026 Sep 30:2026.09.28.755183. Preprint. [Version 1] doi: 10.64898/2026.09.28.755183 (PMC13648636; doi:10.64898/2026.09.28.755183)
Supplement: Supplementary file 5 [file media-5.pdf]

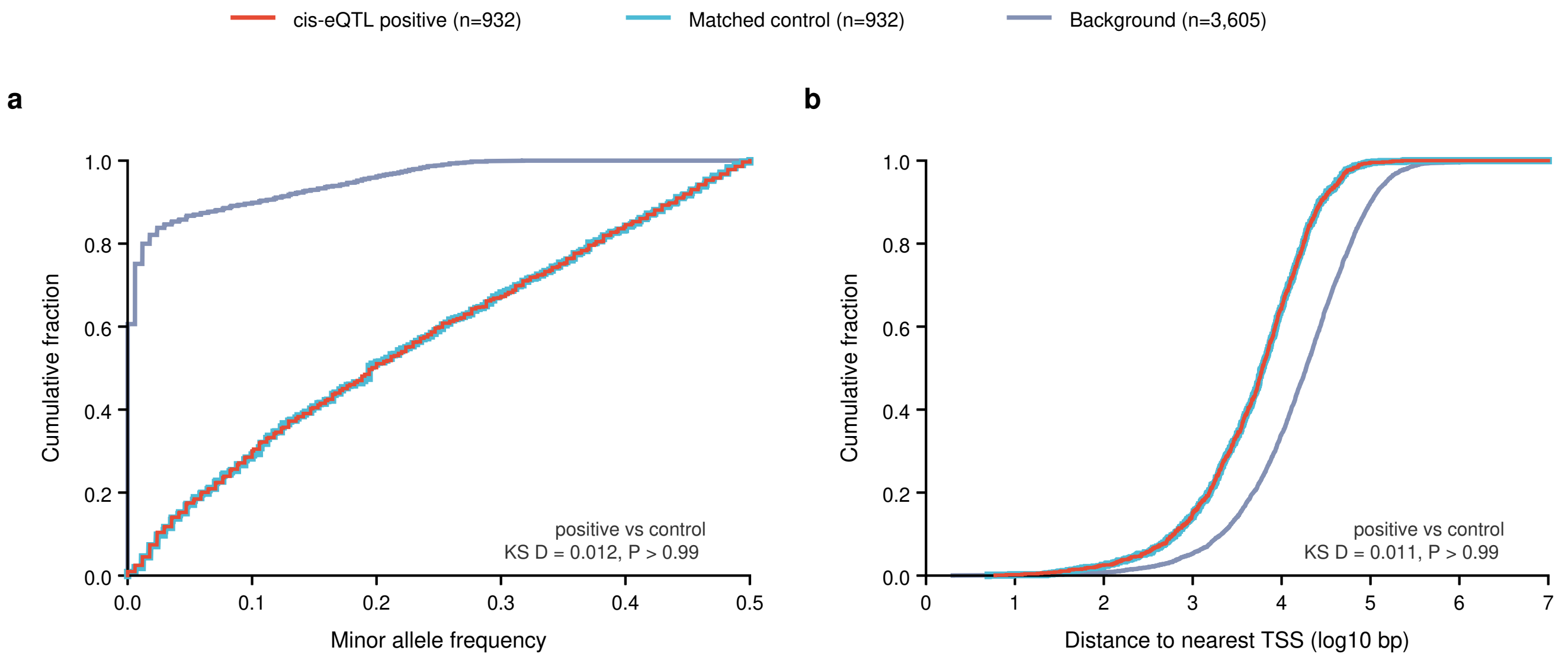
cis-eQTL positive (n=932)
Matched control (n=932)
Background (n=3,605)
a
Cumulative fraction
1.0
0.8
0.6
0.4
0.2
0.0
0.0
0.1
0.2
0.3
0.4
0.5
Minor allele frequency
positive vs control
KS D = 0.012, P > 0.99
b
Cumulative fraction
0
1
2
3
4
5
6
7
Distance to nearest TSS (log10 bp)
positive vs control
KS D = 0.011, P > 0.99
