# Supplementary material for: Mendel, a foundation model of human genetic variation, prioritizes regulatory variants and improves gene expression prediction
Source: bioRxiv. 2026 Sep 30:2026.09.28.755183. Preprint. [Version 1] doi: 10.64898/2026.09.28.755183 (PMC13648636; doi:10.64898/2026.09.28.755183)
Supplement: Supplementary file 6 [file media-6.pdf]

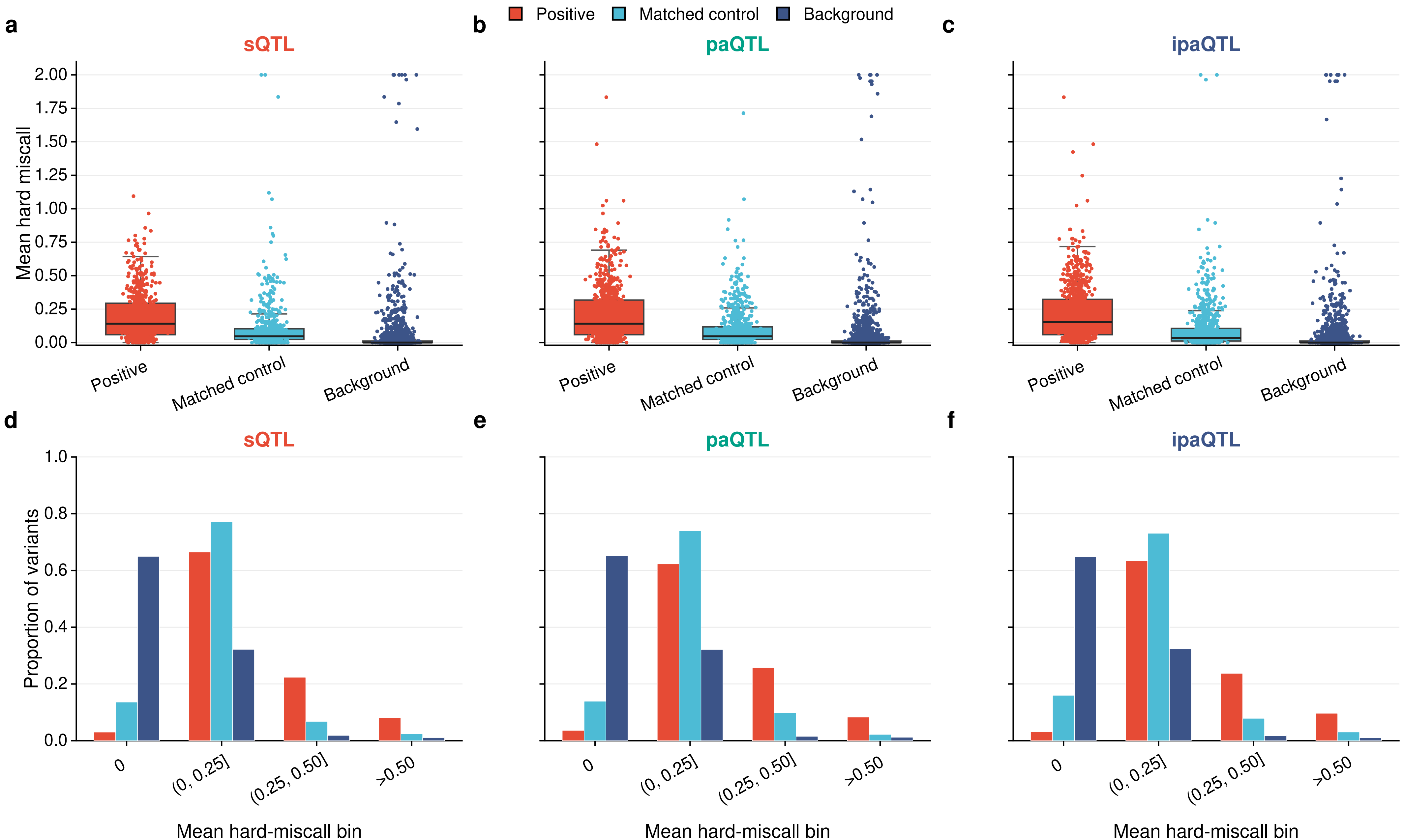
Positive
Matched control
Background
a
sQTL
b
paQTL
c
ipaQTL
Mean hard miscall
2.00
1.75
1.50
1.25
1.00
0.75
0.50
0.25
0.00
Positive
Matched control
Background
d
sQTL
e
paQTL
f
ipaQTL
Proportion of variants
1.0
0.8
0.6
0.4
0.2
0.0
0
(0, 0.25]
(0.25, 0.50]
>0.50
Mean hard-miscall bin
