# Supplementary material for: Mendel, a foundation model of human genetic variation, prioritizes regulatory variants and improves gene expression prediction
Source: bioRxiv. 2026 Sep 30:2026.09.28.755183. Preprint. [Version 1] doi: 10.64898/2026.09.28.755183 (PMC13648636; doi:10.64898/2026.09.28.755183)
Supplement: Supplementary file 7 [file media-7.pdf]

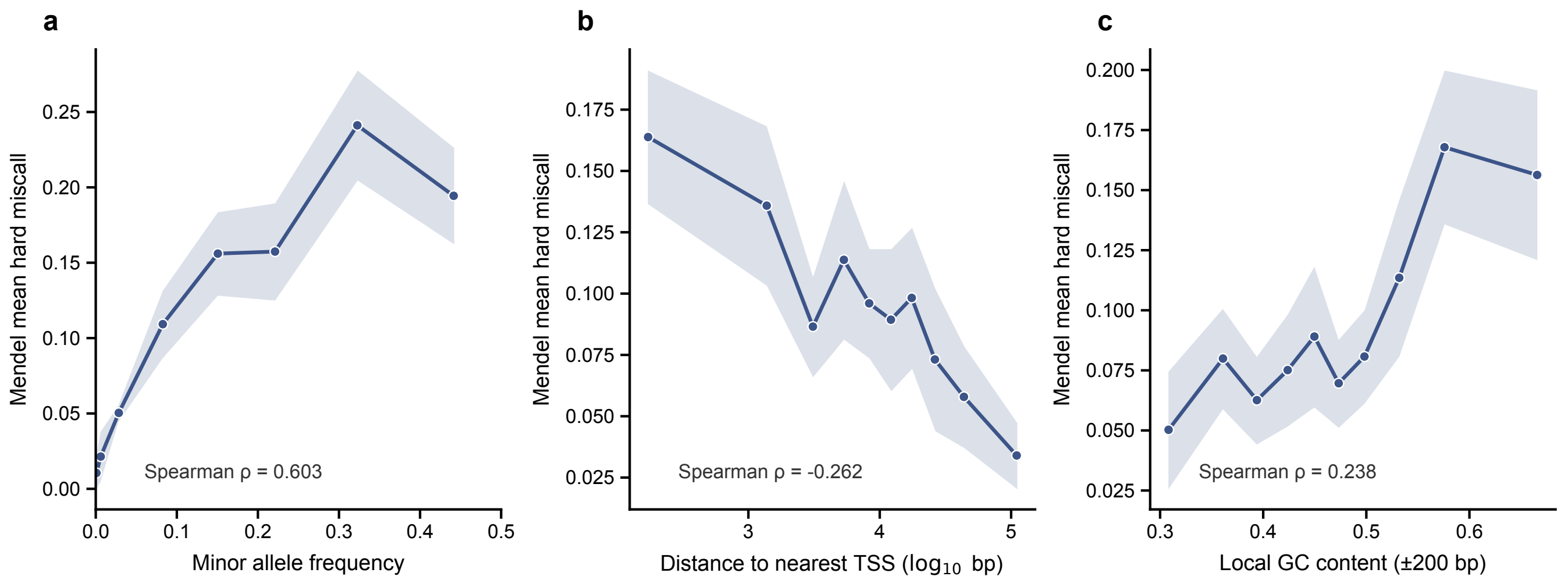
a
Mendel mean hard miscall
0.00
0.05
0.10
0.15
0.20
0.25
0.0
0.1
0.2
0.3
0.4
0.5
Minor allele frequency
Spearman ρ = 0.603
b
Mendel mean hard miscall
0.025
0.050
0.075
0.100
0.125
0.150
0.175
3
4
5
Distance to nearest TSS ($\log_{10}$ bp)
Spearman ρ = -0.262
c
Mendel mean hard miscall
0.025
0.050
0.075
0.100
0.125
0.150
0.175
0.200
0.3
0.4
0.5
0.6
Local GC content (±200 bp)
Spearman ρ = 0.238
