# Supplementary material for: Mendel, a foundation model of human genetic variation, prioritizes regulatory variants and improves gene expression prediction
Source: bioRxiv. 2026 Sep 30:2026.09.28.755183. Preprint. [Version 1] doi: 10.64898/2026.09.28.755183 (PMC13648636; doi:10.64898/2026.09.28.755183)
Supplement: Supplementary file 8 [file media-8.pdf]

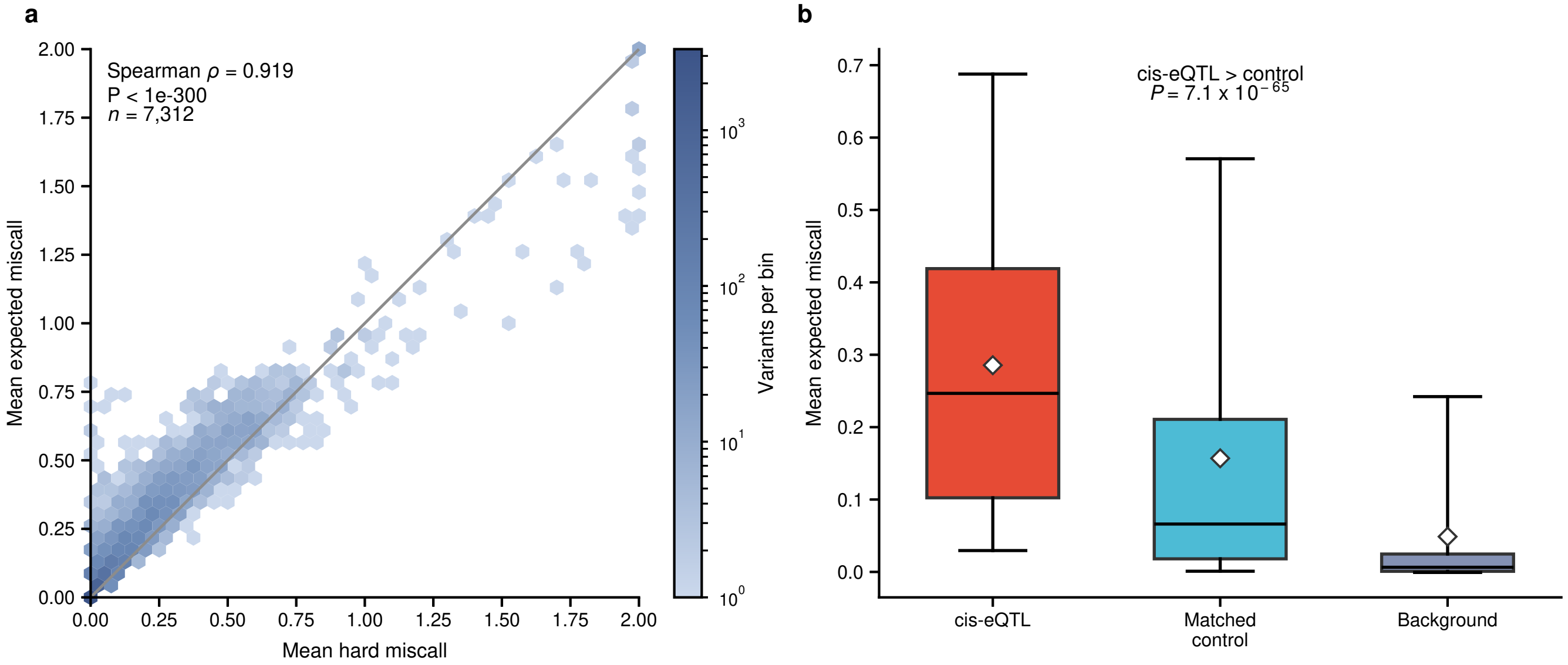

a
Spearman ρ = 0.919
P < 1e-300
n = 7,312
Mean expected miscall
Mean hard miscall
0.00
0.25
0.50
0.75
1.00
1.25
1.50
1.75
2.00
Variants per bin
10^0
10^1
10^2
10^3
b
cis-eQTL > control
P = 7.1 x 10^−65
Mean expected miscall
0.0
0.1
0.2
0.3
0.4
0.5
0.6
0.7
cis-eQTL
Matched control
Background
