# Supplementary material for: Mendel, a foundation model of human genetic variation, prioritizes regulatory variants and improves gene expression prediction
Source: bioRxiv. 2026 Sep 30:2026.09.28.755183. Preprint. [Version 1] doi: 10.64898/2026.09.28.755183 (PMC13648636; doi:10.64898/2026.09.28.755183)
Supplement: Supplementary file 9 [file media-9.pdf]

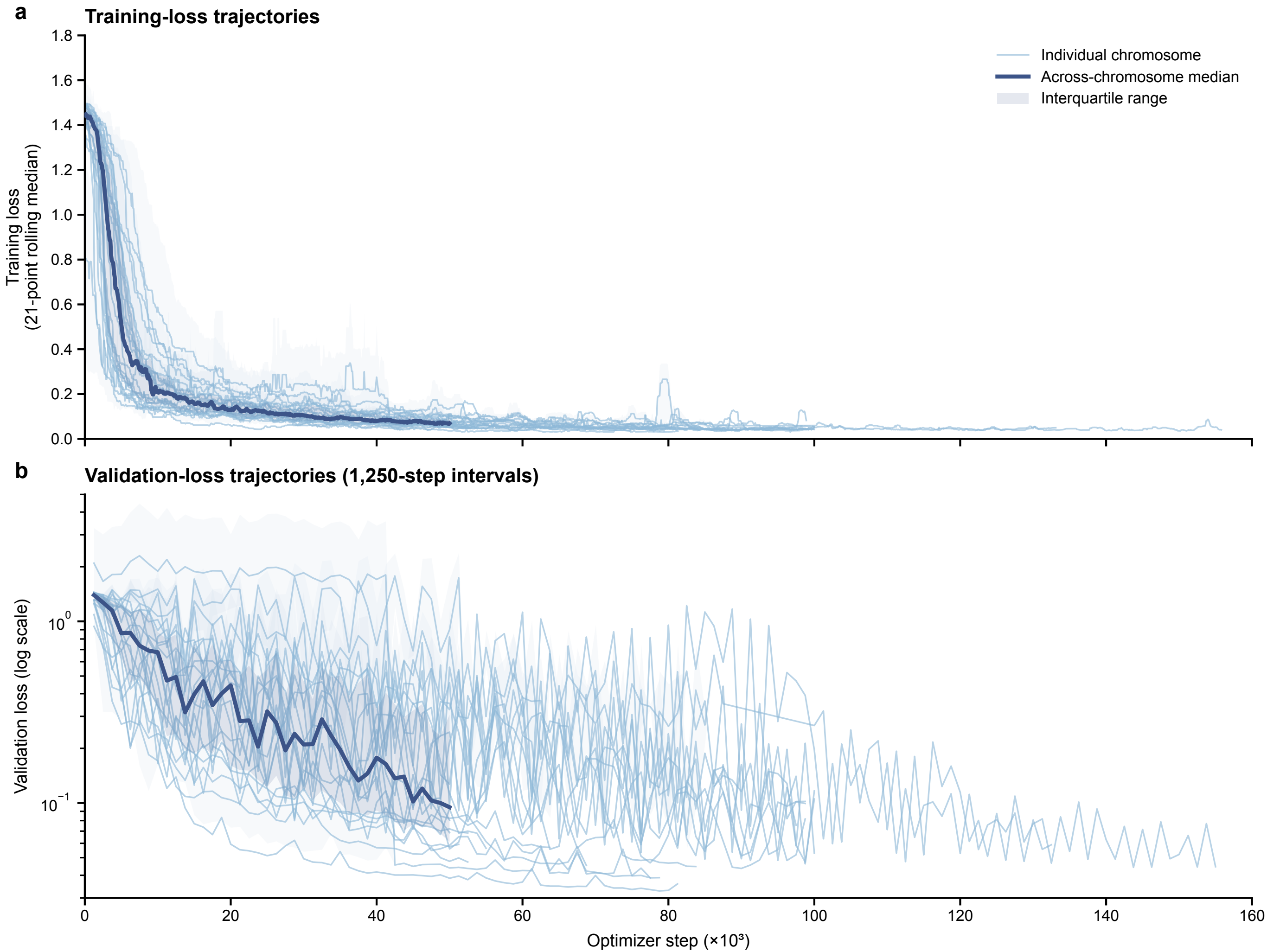

Median and interquartile range are shown through 50,000 optimizer steps, where all 22 chromosome models contribute.
W&B exports dated 20 August 2026; faint ribbons show min–max ranges for grouped chr10, chr11, chr12 and chr22 traces, and one chr14 validation point.
